# Telling is Caring: Prioritizing the Best Interests of Dying Children

**DOI:** 10.1007/s10730-026-09584-x

**Published:** 2026-04-04

**Authors:** Stella Mosetti

**Affiliations:** Interdisciplinary Centre for Health Science, Sant’Anna School of Advanced Studies, Pisa, Italy

**Keywords:** Best interests, Therapeutic privilege, Non-disclosure, Pediatric end-of-life care, Shared decision making, Pediatric ethics

## Abstract

In contexts where children face imminent death despite ongoing medical care, a significant ethical question arises: should they be informed of their approaching death? Although international guidelines now advocate for honest and developmentally appropriate communication, parents and clinicians are sometimes still inclined to withhold this information. Such non-disclosure may be viewed as an exercise of therapeutic privilege, potentially justified by the Best Interest Standard, the prevailing framework for proxy decision-making in pediatrics. Yet it remains contested whether concealing end-of-life information truly aligns with a child’s best interests, as evidence suggests that withholding information may not always serve the child’s overall well-being. This paper critically examines the legitimacy of non-disclosure in pediatric end-of-life care. It explores whether non-disclosure can be inherently beneficial and whether children possess an intrinsic interest in knowing about their imminent death. Moving beyond the dichotomy of disclosure versus non-disclosure, the paper proposes a nuanced, interest-based evaluative framework, where truth is treated not as an absolute value but as a means to promote the child’s overall well-being.

## Introduction

Advances in modern medicine have enabled many children with complex and life-limiting conditions to survive illnesses that were once invariably fatal. As a result, a growing disconnect has emerged between diagnosis and long-term prognosis, and 21 million children worldwide now require palliative care each year (WHO, 2018). The health of these children varies widely: some gradually decline due to accumulating complications, others worsen at first then stabilize temporarily before declining again, and others alternate between stable periods and crises (Linebarger et al., 2022). In the United States, most children and adolescents die in hospital settings, typically following the withdrawal or withholding of life-sustaining interventions, especially in cases involving medical complexity (Linebarger et al., 2022; Meert et al., 2015).

In some cases, death becomes imminent despite ongoing medical care. Since the literature offers no universally accepted definition of “imminent death” (Hui et al., 2014), the term is best understood as referring primarily to a critical clinical turning point rather than to a fixed temporal threshold. Specifically, it denotes the moment at which death is expected to occur in the near future rather than at an indeterminate or distant time, despite a long-standing poor prognosis^1^ (Alsuhail et al., 2020). While no precise timeframe defines this phase, it is commonly described in the literature as extending from a few days to, at most, a few weeks and as reflecting an irreversible progression toward death (Alsuhail et al., 2020).

This delicate phase not only marks a clinical turning point but also raises important ethical considerations, which become particularly pressing once death is both temporally close and clinically irreversible: should children be informed about their imminent death? Historically, healthcare professionals and families have had little evidence-based guidance on how and whether to communicate such information (Stein et al., 2019). However, recent research has enriched our understanding of the psychological perspectives of children, families, and clinicians, and international guidelines now advocate for honest and developmentally appropriate communication (WHO, 2018; Katz et al., 2016). Despite these recommendations, studies suggest that some parents, and at times even physicians and nurses, are still inclined to withhold life-threatening diagnoses from pediatric patients (Cole & Kodish, 2013; El Ali et al., 2024). Although the prevalence of nondisclosure remains uncertain, the ongoing occurrence of this practice (Lövgren et al., 2019; van der Geest et al., 2015; Adduci et al., 2012) encourages an in-depth exploration of the topic from an ethical perspective.

Several rationales have been proposed for nondisclosure. Withholding information could be justified as serving the child’s *best interests* (El Ali et al. 2024) through therapeutic privilege^2^ (Shalak et al., 2022), aiming to reduce emotional distress and preserve hope (van Straaten, 2000; Martinez et al., 2021). Although therapeutic privilege is conventionally understood as a component of medical ethics exercised by physicians under specific circumstances, parents may also regard the withholding of truth from their children as morally justified to protect them from further emotional harm. Accordingly, they may ask physicians to limit or adjust the disclosure of information (Taub et al., 2023), also influencing how therapeutic privilege is applied in clinical practice. Indeed, while physicians and parents have different roles, parents’ role carries increasing weight in decision-making (Hudson et al., 2019).

Children are often seen as very fragile beings, and their young age can lead to assumptions about their ability to understand what is happening. Yet, children are unique individuals with diverse experiences, and what constitutes their “best interests” is deeply contested. Their developing capacity to direct their own lives makes them particularly vulnerable (Gillam et al., 2022; Krutzinna, 2022), and decision-makers often rely on generalized assumptions rather than the child’s individual needs (Krutzinna, 2022; Bartolome 1981). Focusing on their emotional protection risks overlooking broader interests, including relationships with caregivers, understanding and making sense of life, and participating in decisions about their care (Gillam et al., 2022; Krutzinna, 2022).

Unlike authors such as Gillam et al. (2022), who emphasize truth-telling as a foundation for children’s future autonomy, this paper focuses on children facing imminent death, for whom no such future exists. Here, the ethical significance of disclosure lies in children’s immediate psy parents and medical personnel — determine whether to communicate imminent death. Disclosure is not about preparing children for later decisions, but about safeguarding present interests, enabling self-expression, understanding, and relational closeness in their final days.ychological well-being, their capacity to process the end of life, and the preservation of meaningful relational moments. While this paper does not explore children’s active involvement in decision-making, it remains child-centered by examining how moral decision-makers — mainl

Using the Best Interest Standard (BIS) and the ethics of therapeutic privilege, this paper develops a critical framework for evaluating truth-telling at the end of life. Section 2 presents two clinical cases; Sect. 3 examines the ethical and practical responsibilities of physicians and parents in disclosing medical information to children; Sect. 4 analyzes the Best Interests Standard and therapeutic privilege in pediatrics; Sect. 5 reviews empirical evidence on the consequences of nondisclosure; and Sect. 6 addresses the role of discretion in communication. Building on Gillam et al.’s (2022) “cluster of interests” model and Malek’s (2009) framework, the paper argues that truthful communication in cases of imminent death can protect a broad range of children’s interests and promote their well-being. It ultimately supports disclosure as the default approach, while acknowledging exceptional cases where information may need to be limited, and it concludes by recommending a shared decision-making framework to guide disclosure decisions with children.

## Therapeutic Privilege in Pediatric End-of-Life Care: Clinical Cases and Key Issues

End-of life in pediatric care raises significant issues regarding communication and decision-making. A clinical case reported by Hollander et al. (2016) reflects this complexity.

An 11-year-old child with a history of heart transplantation developed severe heart failure, and he was treated with a biventricular VAD. The condition of the patient worsened due to complications such as stroke, renal failure, and respiratory failure. Despite various interventions, the child’s health did not improve, and he was removed from the transplant list. After extensive consultation with the family, the palliative care team and the ethics committee, it was decided to discontinue VAD support and allow death to occur, without informing the patient of the plan to avoid causing unnecessary anxiety.^3^  

Ventricular assist devices (VADs) provide partial or complete support to the failing heart and are increasingly used as destination therapy^4^ (Melendo-Viu et al., 2023; Vis et al., 2022). Living with a VAD entails multiple burdens, including comorbidities and adverse events (Jezovnik et al., 2017; Pae et al., 2007). Compassionate deactivation (Hollander et al., 2016) (CD) occurs when burdens outweigh benefits, and the device is withdrawn. This case is particularly significant because the decision to perform CD while the child is conscious is rare. Ideally, advance care planning begins early and involves the child in shared decision-making with their parents (Linebarger et al., 2022). In practice, several barriers limit this process, including physicians’ assumptions about parental “readiness” and concerns that disclosure might undermine hope (Durall et al., 2012; Sanderson et al., 2016).

Another case, reported by Taub and colleagues (2023), seems to propose a similar scenario.

A 7-year-old has idiopathic pulmonary hypertension diagnosed 1 year ago. The patient is on maximal medical therapy and is not a transplant candidate. The child has done an amazing job adapting to the current circumstances, including the need for continuous subcutaneous treprostinil. But, of late, the child’s clinical condition has deteriorated, with more extreme exertional dyspnea. The parents recognize the inevitability of their child’s impending death and have consented to hospice enrollment. The parents request that the team not share the prognosis with their child, noting the child’s young age and lack of maturity to understand and cope with that news.

In this case as well, it is evident that the emphasis has shifted from curative interventions to optimizing the child’s comfort and remaining quality of life. In both instances, the parents act as the primary decision-makers, choosing to withhold prognostic information from the child. Parents, as proxies, have a primary role in such communication, as their role has profound implications for how professional communicate with seriously ill children (Linebarger et al., 2022).

As we read these cases, some questions inevitably arise. Would it have been morally appropriate to inform the child that his VAD was being deactivated? Was it in a seven-year-old child’s best interests to know the truth about the end of his life? Who should have had the role of communicating the truth to the child? The answers to these questions involve various ethical aspects. Some of these aspects concern strictly moral considerations, such as whether lying or withholding information is inherently morally right or wrong, or whether it is right to exclude children from decisions about the end of their life. Empirical research can inform other aspects, for instance, by exploring whether withholding significant information from children benefits them, or what children know about death.

Such findings are directly relevant to the practice of therapeutic privilege, which involves withholding information when it is in the patient’s best interest (Shalak et al., 2022). While this practice is typically associated with physicians, parents may also choose not to fully disclose information to their child. Despite its significance, this phenomenon remains relatively underexplored in cases of imminent pediatric death. A recent review found that only a few studies specifically address the practice of withholding information during this phase (El Ali et al., 2024). This relatively modest focus in the literature may be explained by two factors.

On one hand, there is limited documentation on how often children are not informed about their future death. Nevertheless, there are some studies indicating that this practice is still carried out, by both parents and physicians, despite a gradual shift toward an open approach with children over the years. For instance, a study (Lövgren et al., 2019) that examines end-of-life conversations in Sweden for children aged 0 to 17 who died between 2015 and 2017, using data from the Swedish Palliative Care Registry, indicates that fewer than half of the children had an end-of-life discussion with a doctor^5^. Another study found that 86 parents (64% in the study) in the Netherlands did not address the forthcoming death with their child (1–17 years old) (van der Geest et al., 2015). Research from Italy investigating communication between parents and children with cancer showed that 12 of 64 children aged 4–18 years didn’t receive information about their disease (Adduci et al., 2012).

On the other hand, as noted, there is a genuine conceptual problem in defining what constitutes “imminent death”. Beyond its use for the purposes of this paper, the lack of standardized definitions for key terms in palliative care, such as “end of life” and “terminally ill patient”, highlights how ambiguity in clinical language can generate uncertainty among physicians and patients, complicating research and therapeutic decision-making (Hui et al., 2014). Regardless of diagnosis, a patient’s disease trajectory may sometimes be predictable; in other cases, it may involve a slow decline with varied symptoms, following a course marked by considerable unpredictability (Linebarger et al., 2022). Given this uncertainty, it is also questioned when it would be appropriate to communicate this news to the child. Moreover, physicians may sometimes overestimate actual survival time (Higginson, 2002). As some authors rightly suggest, end-of-life care and palliative care often overlap in managing individuals with long-standing illnesses, such as cancer (Lundquist et al., 2011). Given that “imminent death” is defined here as roughly the last few weeks of life, it is notable that physicians’ predictions are generally most accurate during this period, particularly during the final two weeks (Christakis et al., 2000). This alignment between definition and clinical predictability reinforces the practical relevance of using the last weeks as a reference point for imminent death.

## Disclosure Responsibilities and Ethical Perspectives in Pediatric Care

In addition to the difficulties previously outlined, other issues arise in such scenarios, particularly regarding ethical perspectives on disclosure, both with respect to the role of physicians and that of parents.

### The Role of Physicians

The concept of therapeutic privilege is primarily applied to physicians, who may, in certain circumstances, decide not to disclose the truth (or the whole truth) to a patient. This discretion is exercised with the intent of preventing harm to the patient, based on the physician’s professional judgment and medical expertise. However, there are different perspectives on this practice. Some scholars believe that therapeutic privilege, especially in the case of competent patients, is generally wrong (Edwin, 2008), also because it damages the therapeutic relationship between the doctor and the patient; others argue that it could be justified in specific situations (Shalak et al., 2022; Kling, 2012). Beauchamp and Childress (2013) consider therapeutic privilege to be “uncontroversial” in at least three circumstances: emergencies, incompetence, and waiver (p. 127). They nonetheless acknowledge that any attempt to justify therapeutic privilege ultimately rests on the principles of beneficence and nonmaleficence, insofar as nondisclosure is intended to promote the patient’s good (p. 128). Within this same ethical framework, benevolent deception can also be situated. Although it is regarded as more controversial, it involves an assessment of the risks and benefits that the practice may entail for the patient’s overall well-being. In this regard, the authors also note that veracity is a *prima facie* obligation rather than an absolute one (p. 303), and they argue that the most appropriate approach is often to disclose information gradually over time. They further recognize that, in some circumstances, full disclosure may undermine a patient’s hope, particularly when recovery is expected (p. 306).

In the context of pediatric end-of-life care, benevolent deception appears to play a key role, also because in ethical decision-making concerning children minimizing distress is a primary concern. In addition, children’s young age may lead to assumptions about their limited understanding of their medical condition. However, young children are often able to understand health-related information when it is tailored to their developmental level (Yuan et al., 2021), and, for this reason, honest communication is recommended and adapted according to their age and development (Bell et al., 2016). Despite this, children sometimes report that they do not receive enough information related to their health, and this may lead to missed opportunities in expressing their opinions (Coyne & Yuan, 2025). In such contexts, benevolent deception may play a predominant role, especially when clinicians prioritize emotional protection over transparency. Healthcare personnel may, at times, assume a protective role toward the child, and some studies highlight that this practice can manifest as an act of omission of difficult news (El Ali et al., 2024).

### The Role of the Parents

While the concept of therapeutic privilege is traditionally applied to physicians, it highlights a broader ethical phenomenon: the discretionary authority to withhold information for the perceived benefit of the patient. Parents, by virtue of their responsibility and authority over their children, may similarly hold a privileged role in decision-making. Pediatric practice increasingly recognizes that physicians are not the sole decision-makers; parents are now acknowledged as central participants in care, both in sharing information and in providing emotional support to the child (Hudson et al., 2019), bringing knowledge and perspectives that the physician may not have. Their role involves guiding choices based on family authority, values, cultural norms, and personal priorities, all of which are essential in determining what constitutes a “good” decision for the child’s quality of life (Adams et al., 2017). Considering these complex decision-making situations, parents may sometimes request that the prognosis not be disclosed (Taub et al., 2023), while physicians may struggle to communicate it to the parents, mitigate its impact (Porter et al., 2023), or defer to the parents’ wishes, acknowledging their authority over the child (Taub et al., 2023). Even when parents request nondisclosure, their underlying goal is often similar to that of therapeutic privilege: to act in the child’s best interest, protecting them from the distress of knowing their condition or from information they may not yet be able to fully comprehend. (Taub et al., 2023). For parents realizing that their child is dying is profoundly distressing, and parents of children with life-limiting conditions may feel they have failed in their protective role (Bluebond-Langer, 1978). Building on this, research on children with medical complexity shows that parents’ beliefs about what it means to be a “good parent” directly shape the decisions they make on behalf of their child (Jonas et al., 2022). Many parents interpret being a good parent as protecting their child from anxiety or emotional distress, which can lead them to withhold or filter information they perceive as potentially harmful. Linebarger and colleagues (2022) further highlight that such beliefs are explicitly considered important in pediatric advance care planning and can guide decision-making in this context. Taken together, these studies suggest that parents’ decisions to withhold information are often guided by a sincere desire to protect their child from emotional distress, reflecting their personal ideals about parenthood. These beliefs can, in turn, significantly influence the final decision-making process.

### Towards a Shared Decision Making

Beyond the ethical considerations that may lead a physician or a parent to withhold the truth from children, there is also an inherent challenge: the literature often fails to clarify who should take on the difficult role of disclosing the child’s condition and its severity (Miller et al., 2022). The lack of clearly defined responsibilities in this context can create barriers to disclosure, as some professionals (such as nurses, oncologists, or other physicians) may feel that communicating the truth “is not their job” (Miller et al., 2022). Parents, in turn, sometimes report feeling left alone during critical moments in palliative care, particularly regarding communication practices (Durall et al., 2012). Furthermore, there are frequently “gaps” between what the clinician perceives as the best course of action and what the family values most. While physicians tend to focus on clinical effectiveness, families prioritize the child’s lived experience (Adams et al., 2017).

The three parties involved in these decision-making processes, namely the parents, the child, and the medical team, appear to have distinct roles. Typically, the physician uncovers the prognosis and communicates it to the parents. The child may sometimes be present during these discussions, but at other times they are not (Boeriu et al., 2023). In such cases, a proxy decision-making scenario arises, in which someone must decide whether or not to convey the information to the child. This situation can leave physicians and parents with the responsibility of determining how much information to share, creating a complex dynamic that raises important ethical questions about how decisions can best reflect the child’s interests. At this stage, conflicts may also arise, as parents and physicians can have different perspectives on the child’s best interests, and conflicts in pediatric decision-making regarding this issue are well known (De Sabbata & Pearson, 2024).

In response to these challenges, shared decision making (SDM) has received increasing attention in recent literature as a structured, collaborative approach to addressing complex healthcare decisions. Although this aspect will be discussed in greater detail, a provisional definition of this approach in this context is that it is an interactive process in which patients (including families and children) and physicians (along with other relevant professionals) actively participate in all stages of decision-making and jointly develop a (treatment) plan for implementation. Several key conditions characterize this process: (1) the involvement of at least two parties, (2) a two-way exchange of information, (3) a shared understanding of the available treatment options and their implications, and (4) equal consideration of the knowledge and value-based priorities each party brings to the decision-making process (Adams et al., 2017). Ultimately, SDM aims to guide decisions that balance medical expertise with family values and preferences, ensuring that the child’s best interests remain at the center.

## The Role of the Best Interests Standard

The ethical inclination to avoid discomfort in children is embedded in a framework that evaluates medical treatments according to the child’s best interests. The Best Interests Standard (BIS) has been the prevailing ethical principle in pediatric ethics for decades (Bester, 2019) and is frequently invoked in discussions ranging from the ethics of childcare (Birchley, 2021; Parker, 1994) to international law. Article 3 of the United Nations Convention on the Rights of the Child (CRC) (United Nations, 1989) states that “In all actions concerning children (…) the best interests of the child shall be a primary consideration”.

Philosophically, interests are defined broadly. Wilkinson (2006, p. 455) describes an interest as “having a stake in something—that is, standing to gain or to lose, depending on the nature or conditions of that something”. In the medical context, interests correspond to what respects a person’s well-being, or “what makes a person’s life go well” (Gillam et al., 2022). A subject may possess interests independently of their ability to assert them. For instance, non-human animals may have an interest in not suffering, even though they are unable to articulate that interest (Singer et al. 2022). While children are not non-human animals, they similarly possess interests that may not always be articulated. For this reason, someone takes on the responsibility of giving voice to such interests. Several factors are commonly considered in clinical practice when determining what constitutes a child’s interests. Such elements could include the considerations of the child’s health status and general well-being, considerations regarding the quality of life for both the child and the family and how current decisions may affect the child’s future.

More difficult, instead, is determining which of these interests constitute the patient’s *best* interests. Often, determining a child’s best interests implies a cost-benefit evaluation, and for this reason in the bioethics literature the concept of best interests is commonly associated with consequentialism^6^, which is understood to provide the theoretical foundations of this perspective (Birchley, 2021). Indeed, it is supposed that BIS could help in the proportional weighing of the benefits and burdens borne by patients, with regard to various treatment options, and that it should promote the maximal good of the individual (Buchanan and Brock, 1990; Passos Dos Santos et al., 2023).

However, while BIS continues to guide clinical decision-making, especially in the absence of explicit guidelines, it remains subject to debate. The term “best interests” is inherently ambiguous, as determining what is right (ethically appropriate), good (morally or personally beneficial), or optimal (maximizing overall well-being) for an individual often involves significant normative and contextual complexity typical of proxy decision-making scenarios (Comerci et al., 2026). Critiques highlight vagueness, referring to the lack of clear criteria for determining what factors constitute the patient’s best interests (Veatch, 1995); individualism, pointing to the assumption that what is best can be defined in isolation from broader social, familial, or cultural contexts (Diekema, 2004); and potential self-defeat, meaning that, although the standard is intended to benefit the patient, its application by a clinician can inadvertently reduce the patient’s overall well-being by assuming the professional knows better than the patient what is truly best for them (Veatch, 1995). Children’s developing capacity for autonomy further complicates assessments (Gillam et al., 2022; Krutzinna, 2022), and reliance on generalizations or category-based assumptions can obscure their individual needs (Krutzinna, 2022). Moreover, acute care often emphasizes generalized pediatric outcomes over the values of individual children (Bartholome, 1981), underscoring the need to integrate BIS with complementary principles and models for a more balanced approach.

### The Individual Child and the Child as a Collective

A significant contribution in this regard is Krutzinna’s Model of the Individual Child (MIC) (2022) which introduces a differentiation in the assessment of children’s BIS. While authors as Buchanan and Brock (1990) argue that decisions for children typically emphasize objective developmental conditions rather than current preferences, Krutzinna’s argument is based on the fact that the BIS still lacks a “robust understanding” of *who* is the child, beyond generalizations about children’s needs in decision making.

While a child may be legally defined as any individual below the age of majority (a threshold that varies across jurisdictions) children have often not been recognized as a category in their own right, but rather defined in relation to, and in contrast with, the category of “adult”. This relational and capacity-sensitive understanding of childhood supports the widely accepted view that adults and children appropriately differ in the kinds of actions and decisions they are capable of undertaking. Children’s more limited decisional capacities, which reflect their stage of developmental maturity, do not constitute a deficiency in themselves but rather represent a normal and expected feature of childhood. Accordingly, children are commonly regarded as vulnerable insofar as they appropriately rely on others for support and protection, given their not yet fully developed capacity for autonomous decision-making (Gordon, 2020). This perspective reflects a categorical approach to vulnerability (Macklin, 2003), according to which all children are considered vulnerable by virtue of shared developmental characteristics. In bioethics, several approaches to vulnerability exist; however, vulnerable individuals are generally regarded as deserving a higher degree of protection than non-vulnerable individuals, raising crucial questions about the nature and scope of such protection.

Although a categorical approach might suggest that, because children are primarily viewed as recipients of protection, it is preferable to withhold painful truths from them, broad assumptions about vulnerability can complicate the assessment of a child’s best interests. A general approach alone does not justify non-disclosure. Instead, recognizing a child’s vulnerability should prompt careful consideration of what serves their best interests in their specific circumstances. Protective measures may be necessary to help vulnerable individuals exercise their rights, but the mere intention to protect does not automatically ensure that these measures promote well-being.

Moreover, a categorical approach may overlook the fact that children often belong to multiple vulnerable categories, each requiring attention to their specific needs. For example, children with complex medical conditions are frequently described in clinical literature without clear, universally accepted criteria (Gallo et al., 2021), which can result in their needs being overlooked or misunderstood. This population is understudied, and their specific vulnerabilities and subjective experiences are often insufficiently considered in clinical decision-making. A medically complex child may have vulnerabilities not shared by a non-medically complex child, and vice versa. Thus, a categorical approach may lack the nuance required for effective safeguarding, whereas a contextual approach enables more targeted and precise interventions.

Assumptions about vulnerability can also foster implicit biases regarding what is considered appropriate or beneficial for a given category of individuals. Similar concerns have been observed in other areas of medical ethics, where external observers may underestimate the quality of life of individuals facing severe illness or impairment (Campbell et al., 2021). While children should not be equated with persons with disabilities, this literature highlights a broader epistemic risk: the tendency to project normative assumptions about suffering, dependence, or diminished functioning onto others. In pediatric decision-making, such projections can lead to misjudgments about what truly serves a child’s interests and contribute to forms of epistemic injustice, particularly when the child’s perspective (or the possibility of accessing it in developmentally appropriate ways) is overlooked or undervalued. This can occur even when actions are motivated by a genuine desire to help or protect.

To address these limitations, Krutzinna’s child-centric model identifies three spheres of the child: the “universal child” the “categorical child”, and the “individual child”. This framework allows practitioners to identify and balance needs shared by all children, needs specific to particular groups, and interests unique to individual children. Discretion plays a central role in avoiding neglect of intra-group differences, including variations in abilities, social categories, and vulnerabilities. Such flexibility is crucial in end-of-life care, ensuring that no dimension of the child’s identity is overlooked. Although Sect. 6 will discuss the role of discretion in considering children’s interests, this child-centered approach ensures that each child’s unique needs are taken into account.

### Limits and Applications of the Best Interests Standard

Building on this framework, the application of BIS to children facing imminent death must carefully balance the child’s individual experiences with ethical and clinical considerations. When considering the child’s best interests in the context of imminent death, non-disclosure could be justified by the belief that a child’s psychological pain, anxiety, and fear upon learning of their impending death outweigh any moral duty to disclose the truth. From this perspective, informing the children about their end-of-life may not serve their best interests, especially given their limited remaining time and the assumption that trust in the therapeutic relationship will remain intact. This reasoning likely underlies the decision not to inform the children discussed at the beginning of this paper.

But are these assumptions truly justified? I would argue that they are not. Relying solely on this reasoning risks overlooking significant aspects of the child’s individual experience and may undermine the nuanced discretion emphasized in Krutzinna’s model. While withholding the truth may be motivated by benevolence, it does not necessarily serve the child’s personal interests. Moreover, there is no consensus in bioethics on whether death itself can constitute a child’s best interest (Savulescu, 2017), or whether BIS should prevail over a child’s emerging autonomy (Ke, 2023; Ross, 2019). Additionally, although some could argue that the therapeutic relationship cannot be harmed if the child is unaware of the truth, as will be discussed later in this paper, children often know much more than was previously assumed. This insight challenges the claim that withholding information leaves the therapeutic relationship unaffected.

Before discussing how these considerations can be integrated into clinical practice, some clarifications are necessary. First, the limitations of BIS pertain to proxy decision-making in general and often cannot be fully resolved (Comerci et al., 2026). When making decisions on someone else’s behalf, there is always a margin of risk that the choice could be wrong, and a certain degree of arbitrariness is inherent in the decision itself. Since children and preadolescents are not considered mature minors capable of independent healthcare decisions (Shalak et al., 2022), legal responsibility lies with parents (Cole & Kodish, 2013), who are often assumed to be the ultimate experts on what is best for their child. However, potential conflicts may arise regarding the interpretation of a patient’s best interests, particularly between parents and physicians. Parents are naturally those most invested in their child’s well-being and typically know their child better than anyone else. At the same time, clinicians may feel a professional duty to communicate the truth to the child. This tension between parental insight and clinical duty underscores the need to clarify whose interests are ultimately at stake. Importantly, the child (not the parents or the physicians) is the ultimate bearer of these interests. The claim to objectivity of BIS, which functions as both a medical and legal standard, must integrate the perspectives of all parties involved but also transcend them when there is objective evidence that a particular course of action would better promote the child’s welfare.^7^

Although the principle of BIS has been criticized, it also has its defenders. For example, Kopelman (1997) notes that BIS should be understood as a “standard of reasonableness” implying a practical decision among several options. This standard “may be less than ideal, but it is often better than a barely acceptable minimum”. Recognizing the limitations of BIS does not justify abandoning it, particularly when children’s preferences and experiences are difficult to ascertain or subject to change. In cases of imminent death and truth disclosure, it is possible, in principle, to explore whether therapeutic privilege could be beneficial and which interests should be protected. Understanding BIS as a “standard of reasonableness” provides the framework that will guide the discussion in the following sections.

## Is Non-disclosure Beneficial?

Having examined the ambiguity surrounding the Best Interests Standard (BIS), it is crucial to evaluate whether non-disclosure can provide the presumed benefits in practice. However, empirical evidence on this matter remains inconclusive. In the context of imminent death, it is inherently impossible to retrospectively assess the benefit of non-disclosure. Nevertheless, references can be made to the literature on children with medical complexity or those with a terminal illness. One attempt could consist in trying to understand how much children wish to be involved in decisions about the treatments they are going to receive. Unfortunately, the data are conflicting on this point, especially in the case of younger children who are not considered mature minors but are old enough to ask questions and express concerns (Gillam et al., 2022). A study that included also 7-year-old children showed that children with cancer wanted to receive information about their treatment (Coyne et al., 2014), but another research supports the hypothesis that medical care for pre-adolescent children was characterized by their passive role, with very limited engagement in care planning conversations (Levetown, and the Committee on Bioethics, 2008). Yet, children know more than we once believed. While there was once a prevailing belief about the naivety of children — namely “What children don’t know can’t hurt them” (Chester et al., 1991, p. 510) — since the seventies, it has been clear that seriously and terminally ill children are at least aware that something serious is happening to them. Research about children’s capacity and perception reports that children who live with a life limiting condition have witnessed and heard of other children’s death; and that dying children can be aware of their impeding death (Binger et al., 1969; Wolfe, 2004). A child could be aware of “the hushed whispers and discussions among grown-ups and can ascertain that a secret exists that is not to be discussed” (Cole & Kodish, 2013, p.64). In some cases, children construct their own explanations for secrecy, including beliefs that the illness is a form of punishment. Research further indicates that children unaware of their diagnosis can sometimes experience more distress than those who are informed (Cole & Kodish, 2013).

Studies by Waechter (1971) and Spinetta et al. (1973) reported data that support the idea that 6–10-year-old children could be concerned about their illness, and although this concern may not always be openly voiced, the fears and anxieties remain deeply painful and closely linked to the seriousness of the illness. Sometimes, children desire to protect their parents from unpleasant news but are still aware of the seriousness of their condition. For instance, Contro and colleagues (2004, p. 1250) reported a child saying “I am going to die but don’t tell my parents because they won’t be able to take it”. Moreover, Spinetta and colleagues have also shown that increased anxiety is observed in children that are aware of their diagnosis without being informed.

Indeed, children may not always show that they are aware that their chances of recovery are very low or nonexistent (Bluebond-Langner et al., 2010), a behavior often reflecting the dynamics of a phenomenon known as mutual pretense (Bluebond-Langner, 1978). In such situations, both the child, the parents and sometimes medical personnel (Goldsmith & Ragan, 2017) may understand the prognosis, yet they avoid openly communicating it to one another to protect each other from fear and distress (Bluebond-Langner et al., 2010). There has been debate regarding whether this phenomenon is potentially harmful (Binger et al., 1969). The dynamics of pretense between parents and children are a well-established feature of parent–child interactions, typically emerging in play and contributing positively to child development by providing a safe space to explore and regulate emotions. In pediatric end-of-life contexts, however, such parent–child pretense can transform into an “elephant in the room” in which the issue is simultaneously present and acknowledged, yet left unspoken. At a certain point, some scholars argue, this pretense may become unsustainable, as the unarticulated reality imposes both emotional and ethical tensions on all parties involved (Goldsmith & Ragan, 2017). This phenomenon can also affect the doctor–patient relationship. Trust is fundamental in any therapeutic relationship, and when the pretense collapses, the child may feel betrayed or confused, reducing openness and collaboration. Nevertheless, mutual pretense functions as a coping mechanism, and like all coping strategies it should be carefully analyzed and understood rather than judged. Human relationships inherently involve secrets, which can help maintain relational dynamics in adaptive ways. Rather than expressing a value judgment on this practice, it is important to recognize that it can be pervasive in pediatric end-of-life scenarios (Sisk et al., 2016).

However, when viewed as a whole, all these findings suggest that deceiving children’s expectations, when they are aware of the seriousness of their condition, can provoke anxiety and fear; sometimes even greater than if they were told the truth. Non-disclosure, therefore, may inadvertently harm the child. Moreover, Kreicbergs et al. (2004) show that parents who lose a child often regret not having discussed death with them, particularly if the child was old enough to understand. This indicates that therapeutic privilege can also be harmful to parents, highlighting its complex ethical implications.

## On Telling the Truth: The Role of Discretion

Given the profound challenges involved, why must we choose to reveal the truth to a child confronting the end of his life? As already seen, while empirical certainty is elusive, cumulative evidence strongly suggests that non-disclosure may, in many cases, cause greater psychological harm than open communication. Healthcare professionals should explore what children understand, as well as whether, when, and how they should be informed, and offer a supportive environment that encourages truthfulness (Dunlop, 2008). Nevertheless, moving beyond consequentialist reasons, the issue of truth-telling in the context of pediatric end-of-life care raises also normative questions about what children are owed as persons (De Sabbata & Pearson, 2024; Jonas et al., 2022), whether they have a moral or legal right to know the truth, and how their interests should be interpreted and protected.

Some arguments in favor of truth disclosure focus on deontological ethics. For instance, Kantian moral theory claims that telling the truth is a categorical duty, independent of outcomes. Others refer to children’s rights: CRC views access to information as part of protecting children’s rights^8^. However, while rights-based approaches offer valuable normative insights, and constitute the foundation for the protection of children, they may not fully address the complexity of pediatric end-of-life decisions. As Gillam et al. (2022) argue, thinking in absolute terms, such as claiming a universal “right to know”, may not be practically helpful when caring for critically ill children. A more flexible, interest-based approach may offer a better ethical framework. Two types of concerns support this view.

First, there are conceptual issues about what it actually means to “tell the truth” to a child. Truth is not a binary concept, and communication must be tailored to the child’s cognitive development. A study has shown that children aged 2–5 typically lack a clear sense of the finality of death, while those aged 9 and older often recognize it as permanent and irreversible (Nagy, 1948). These developmental differences suggest that honest communication must be appropriate; what Gillam et al., drawing on Higgs (2016) refer to as “speaking truthfully”, rather than simply “telling the truth” (Gillam et al., 2022).

Second, there are normative and relational concerns about children’s preferences; namely, whether they wish to know the truth at all. Respecting a child’s right to know also means respecting their right *not* to know (Gillam et al., 2022). While authors like Beauchamp and Childress justify therapeutic privilege when a patient declines to receive information, applying this principle to children is a complex task: their preferences are often unclear or overlooked. This, of course, also has to do with how detectable or observable these preferences actually are, and to what extent they can be considered stable over time. While it is not unreasonable to expect that children (especially if older) might desire to receive information about their own end of life, addressing this expectation can be challenging. In situations where such preferences are unknown or cannot be obtained, it becomes necessary to evaluate and balance a set of interests that reflect each child’s unique circumstances. In these cases, discretion plays a fundamental role, understood as a space of flexibility (Handler, 1986) that allows decision-makers to adapt general rules and categorical knowledge to the specific and unique needs, preferences, and characteristics of each individual child. As noted earlier in the discussion of vulnerability, treating children primarily as members of a broadly defined vulnerable group can risk obscuring the child’s individual needs and capacities; discretion helps ensure that ethical decision-making remains focused on the unique situation and vulnerabilities of the single child rather than on generalized assumptions about children as a category. A response in these complex cases requires a multimodal approach: one that emphasizes the child’s specific interests over abstract, universal rights. These interests may be subjective, relational, or existential in nature. Although this may raise further challenges, such as how to meaningfully incorporate children’s voices and preferences into care planning, it helps avoid placing an unrealistic burden of autonomy on young patients. At the same time, it preserves the possibility that children may *benefit* from knowing the truth, even if they do not hold an absolute right to receive it. In this context, truth is not regarded as an absolute value, but as a potential means of promoting the child’s overall well-being.

## The Interests of Seriously Ill Children

In the context of imminent death, multiple factors must be carefully considered when seeking to promote the interests of children. Considering the framework of Krutzinna (2022), which was already mentioned, it is evident that these children constitute a distinct subgroup of patients. Although numerous aspects concerning these children remain unknown to us, there are certain elements that they undoubtedly share. Children at the end of their lives are usually seriously ill, medically complex, and particularly vulnerable. In such cases, decisions regarding the end of life are not made by the children themselves but rather are communicated to them by adults acting on their behalf. Exercising discretion in this context requires an attentive engagement with all these factors to ensure that every element is not only carefully considered, but also balanced, with other possible characteristics the child could have. These factors do not only concern the child’s age and developmental level, but also a range of elements that may be particularly significant at the end of their life.

In their article on telling the truth to seriously ill children, Gillam and colleagues (2022) consider a cluster of interests that could support the practice of truth telling within general clinical settings. They argue that, although there is no specific reference about truth telling as the best interests for a child, the practice of truth disclosure relates to many others interests that children could potentially have. Referring to a cluster of interests could also fill the epistemological gap surrounding the concept of best interest *tout court*, which has often been criticized for being excessively vague. The authors elaborate a set of interests taken from Malek (2009), as shown in Table 1. Malek’s analysis includes three different perspectives: that of the CRC; *The Irreducible Needs of Children* by Berry Brazelton and Stanley Greenspan’s; and Nussbaum’s list of human capabilities. Malek and Gillam et al. agree that these interests are not independent from one another and must be understood as a unified whole. In themselves, they do not have a hierarchical structure, although I argue that some interests (such as the interest in life) must naturally be fulfilled before others, so that all the others can be realized. The two lists 

(Table 1) are identical, except for some terminological variations: Gillam and colleagues replace

Malek’s terms’ “identity” and “autonomy” with “social identity” and “sense of control”. This terminological adjustment emphasizes that, even if children are not fully autonomous, they still possess an interest in understanding and influencing their own lives, which is particularly relevant in the context of truth-telling at the end of life. Although a child could not be considered fully autonomous and able to take decisions about his healthcare, this doesn’t mean 

**Table 1:** Cluster of children’s interests according to Malek and Gillam et al.

Malek’s list	Definition	Gillam et al.’s list	Relevant for truth telling in the case of imminent death
Life	To live and to anticipate a life of normal length	✓	X
Health and Healthcare	To have good health and protection from pain, injury, and illness. To have access to medical care	✓	X
Basic needs	To have an adequate standard of living, especially to be adequately nourished and sheltered	✓	X
Protection from neglect and abuse	To be protected from physical or mental abuse	✓	✓
Emotional development	To experience emotion and have appropriate emotional development.	✓	✓
Play and pleasure	To play, rest, and enjoy recreational activities. To have pleasurable experiences.	✓	✓
Education and cognitive development	To have an education that includes information from diverse sources. To have the ability to learn, think, imagine, and reason.	✓	X
Expression and communication	To have the ability to express themselves and to communicate thoughts and feelings.	✓	✓
Interaction	To interact with and care for others and the world around them. To have secure, empathetic, intimate, and consistent relationships with others.	✓	✓
Parental relationship	To know and interact with their parents	✓	✓
Identity	To have an identity and connection to their culture. To be protected from discrimination	“Social identity”	✓
Sense of self	To have a sense of self, self-worth and self-respect	✓	✓
Autonomy	To have the ability to influence the course of their lives. To act intentionally and with self-discipline. To reflect on the direction and meaning of their lives	“Sense of control”	✓

he or she has no interest “to reflect on the direction and meaning of his life”.

**Fig. 1:**
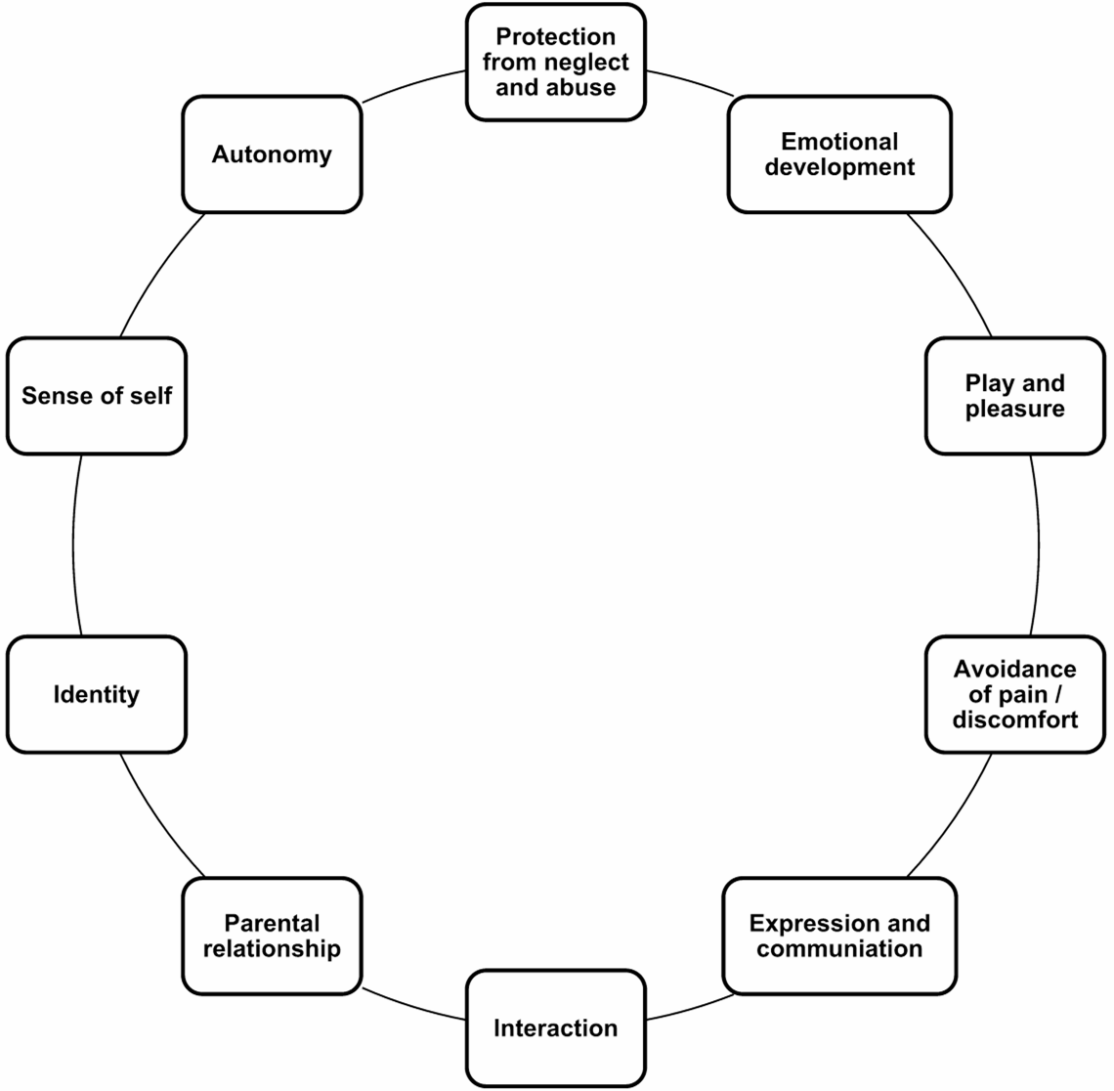
Interests of children at the end of life

Since the authors consider a universal list of interests that should promote the well-being of children, they are not inherently focused on the end-of-life care. Among the two lists, the elementsis paper (Fig.  most relevant to the end-of-life context for a child have been selected for the purposes of the thesis (figure1).

In addition to those included in the table, I also chose to include the issue of the avoidance pain as a separated cluster. Pain or discomfort are particularly significant at the end of life. By avoidance of pain and discomfort, I mean the avoidance of physical pain, as well as of anxiety, fear, and of any form of psychological suffering. Although the question of pain can be included under the umbrella of “healthcare”, as this element appears in Malek’s definition, I argue that this cluster should be kept separate. Having an interest to receive health and health care doesn’t imply inherently the avoidance of pain: for instance, some treatments could be painful, but the child could have interest in receiving them to be cured.

### Case Resolutions

As mentioned at the beginning of this paper, avoiding fear, anxiety, and the distress a child might experience upon learning of their imminent death could be a reason to invoke the therapeutic privilege. However, while the desire to protect children from particularly painful truths about their condition is understandable, relying primarily on the avoidance of psychological pain and fear to determine what is best for the child might not fully reflect all of the child’s needs, as it reflects a rather limited view of the child’s overall interests.

First, because hiding the truth may not necessarily be less psychologically painful for children, who might already be aware of their condition. On the other hand, as Malek (2009) argues, the full promotion of one single interest will not promote the overall well-being of the child. There could also be other interests to consider in the cost-benefit analysis of the practice of truth-telling. One of these factors is the preciousness of the relationship between a child and their parents (De Sabbata & Pearson, 2024) which, for very young children, could be so important that it allows them to endure a certain amount of psychological pain. In addition, the child’s ability to interact with their parents at the end of their life reflects also other interests that a critically ill child could have. For instance, if we consider the child’s interest *in being protected from mental abuse*, and therefore a corresponding interest in “knowing that he or she will be safe and cared for”, omitting the truth may not necessarily help him/her feel that way.

At the same time, the interest in *interaction* makes it important for the child to have consistent relationships with others: an opportunity that would be precluded if such relationships are based on the omission of such significant facts as the end of his life. Moreover, telling the truth to children could satisfy their potential interest in reflecting on the direction and meaning of their life, and so their interest in having a *sense of control*.

Considering the case of the 11-year-old whose VAD support was discontinued, the decision not to inform the child certainly protects the immediate interest of avoiding psychological distress and anxiety. However, it simultaneously limits other significant interests, such as the opportunity to participate in communication about their condition, to develop a sense of control over their life, and to experience meaningful relational moments with their parents during their final days. While emotional protection is important, the ethical framework presented suggests that a child’s overall well-being requires an integrated consideration of all interests: mitigating anxiety alone does not fully respect the child’s “best interests”, whereas carefully supported communication could balance protection with inclusion. Moreover, although disclosing the information may have an emotional impact and potentially affect the child’s remaining days (“emotional development” interest), the child in this condition was already undeniably unable to engage in play as they had before, reflecting a diminished “play and pleasure” interest. It is important to note that describing this interest as “diminished” does not imply that it lacks value in general, but rather that it becomes comparatively less influential in guiding decision-making given the child’s medical complexity and the broader constellation of interests at stake in this specific situation. Moreover, experiencing emotions, even negative ones, is not inherently harmful; on the contrary, it may allow the child to engage in emotional coping and provide an opportunity to express and process these feelings.

Applying the cluster-of-interests approach to the case of the 7-year-old with idiopathic pulmonary hypertension highlights similar ethical complexities. This child is seriously ill and medically complex. The parents have requested nondisclosure of the prognosis to protect the child from emotional distress. From the cluster-of-interests perspective, avoiding fear and anxiety is only one element of the child’s well-being. Other relevant interests include the child’s capacity for expression and communication, interaction with parents and caregivers, sense of control, and the ability to make sense of his experiences. Although age and developmental stage may limit full comprehension, even young children perceive changes in their environment and relationships. Nondisclosure may prevent the child from expressing fears, engaging meaningfully in relational interactions, or gaining a sense of agency in their remaining life. Conversely, sharing the prognosis could provoke distress, but it may also support the child’s understanding, emotional processing, and relational closeness in the final phase of life.

Overall, omitting the truth precludes a range of opportunities aligned with the child’s interests, which extend beyond mere avoidance of pain. Moreover, because experiencing pain is not inherently meaningless, reducing psychological distress does not necessarily equate to acting in the child’s full interest. Enduring the painful knowledge of one’s own impending death can coexist with, and even support, other vital interests, such as maintaining a trusting relationship with parents, being accompanied in final moments, and being reassured that one is not alone. As Gillam and colleagues (2022, p.772) argue:

When the truth is withheld, a child is deprived of the opportunities to grieve and to process, to seek comfort from the supportive relationships that they have, and to develop some sense of control over what is happening. These lost opportunities are a set-back to the child’s interests, even if the child is not aware of the lost opportunity, and does not feel any regret or distress at it.

### Determining Whose Discretion Matters

The crucial question at this stage is how children’s interests should be identified and evaluated, and by whom. A child’s interests may be subject to differing interpretations, and the process of balancing them can diverge between clinicians and parents. Although both parties are generally committed to promoting the child’s best interests, disagreements may arise concerning whether a particular intervention adequately serves one interest rather than another.

In this context, parents occupy a clearly privileged epistemic position. Given their intimate and sustained knowledge of their child, parental input is indispensable for capturing the perspective of the “individual child”. Nevertheless, while the parental viewpoint is unique, it is not absolute. In this sense, guidance provided by a consensus of international pediatric ethics experts is particularly valuable in the development of consensus frameworks for pediatric medical decision-making (Salter et al., 2023). They emphasize that parents should be presumed to have broad but not unlimited discretion in making health care decisions for their children. Furthermore, clinicians are advised to seek state intervention, in addition to complying with mandatory reporting requirements, only when all less restrictive alternatives have been exhausted and a parental decision either places the child at significant risk of serious and imminent harm or fails to meet the child’s basic interests. Crucially, the recommendations highlight that clinicians and parents should collaborate through a shared decision-making process aimed at promoting the child’s overall interests.

As previously discussed, shared decision-making (SDM) represents a particularly promising approach for assessing and balancing these competing interests. SDM enhances a collaborative partnership between clinicians and parents, and, when appropriate, the child, allowing the child’s unique clinical circumstances, developmental stage, and preferences to be meaningfully incorporated into decision-making. This approach is especially valuable in pediatric end-of-life care, where uncertainty and value-laden judgments are pervasive. Recent evidence suggests that, although SDM remains underutilized in pediatric palliative care, it holds significant potential for improving the quality and transparency of decision-making processes (Cai et al., 2023). More specifically, the literature describes two principal models of SDM, which differ in the degree of clinician guidance and parental involvement in deliberation and final decision-making (Cai et al., 2023): the recommendation model and the free market model. The former is primarily adopted for therapeutic decision-making, in which healthcare professionals directly present the option they consider to be in the child’s best interests in order to obtain family consent. The latter is used for decisions not directly related to medical treatments. In this case, the healthcare team explains all available options to the family and guides them in weighing the respective advantages and disadvantages. In this model, professionals do not decide on behalf of the family but rather “teach” them how to evaluate the different possibilities according to their own values. This type of model may be useful in these complex decision-making situations. Very often, as noted, parents are guided by certain beliefs about what it means to be a “good parent”. By contrast, what should guide them is how best to protect their child’s interests. In these cases, physicians have the responsibility not only to explain the available alternatives but also to guide families toward honest communication whenever it serves the 

child’s best interests.

### Possible Exceptions

In a vulnerability scenario, as we have seen, it is inevitable to consider that exceptions may exist. While this paper provides a default position on truth-telling in such circumstances, the possibility of discretionary judgment should nevertheless be acknowledged. The ethical justification for withholding information is based on the same concern for the child’s interests that supports disclosure; however, in some specific situations, withholding the truth may better promote those interests than disclosure alone.

It is important to note that the circumstances discussed here are relatively rare and reflect a specific situation that highlights the importance of honest communication throughout the course of the illness. As mentioned, death, even if imminent, is not a surprise or an unexpected event, but a clinical turning point that is inevitable. This reinforces the idea that families should be guided step by step by physicians and that shared decision-making could also actively involve the child.

In the case of a child with a VAD, there are additional considerations that must be taken into account. For example, if it were discovered that the family had never communicated with the child about his illness. Specifically, when a VAD is implanted, guidelines recommend that both parents and the child should be informed that the child may not reach transplant and could die with the device; either due to complications or compassionate deactivation (Lorts et al., 2021). However, we know how pervasive the phenomenon of mutual pretense can be in these contexts, and there may be numerous obstacles to communicating this truth. Mutual pretense is not an isolated act by the patient or the family, but rather a true agreement among care providers, the patient, and the family; rarely made explicit, yet coordinated in a complex manner (Goldsmith & Ragan, 2017). In this case, not knowing what the child understands, and having never informed him of his condition, revealing the truth could feel like a betrayal, compromising the therapeutic relationship and potentially other key interests, such as protection from neglect or harm. Disrupting an already complex family dynamic could also increase the child’s distress, as it would undermine a precarious equilibrium that the child has attempted to construct.

Had the child been gradually informed throughout the process, we might have identified additional aspects of his interests. If children were gradually made aware of the progression of their illness and understood what their parents knew about it, the question of whether withholding the truth might serve the best interests of the patients would be far less likely to arise. Since it has been shown that children can suffer greatly from hiding the truth from their parents, open and honest communication throughout the course of care could help them feel less alone in facing their own mortality.

Conversely, if the child had been informed of their condition, another scenario could emerge: they might explicitly request not to be told certain details. In this case, withholding information would respect several of their interests, including a sense of control over the end of life, trust in their parents, and emotional development. This is supported by reports in which parents stated that their child expressed a desire not to discuss the topic of death (van der Geest et al., 2015). Such situations represent cases in which even the therapeutic privilege might also be ethically justified (Beauchamp & Childress, 2013).

## Limits, Possibilities and Recommendations

At the end of this reflection, it is important to clarify that the approach proposed in this paper does not claim to be a perfect model of discretion. The aim of this paper was not so much to propose a perfect discretionary model, but rather to demonstrate that even children who are not considered autonomous in making decisions about their health and end-of-life matters still have interests that support the idea that telling them the truth is better than withholding it.

Like any ethical framework, this approach inevitably has its limitations; first and foremost, understanding how to effectively balance these interests and why the ones listed should be prioritized over others. However, it must be acknowledged that Malek and Gillam’s et al.’ list is undoubtedly very comprehensive and considers three different perspectives which, taken together, are quite extensive. Secondly, the list could certainly be expanded, but even these elements alone seem to indicate that, even if children may not have an intrinsic interest in the truth, the sum of these interests should encourage truth disclosure, especially when the child is capable of understanding what is happening. It means taking responsibility for one’s actions toward children who are experiencing conditions of extreme vulnerability. For this very reason, more research is needed on this group of children, their wishes, and their perceptions. This would help in making better decisions, even though a certain degree of risk in making moral choices like these is inevitable. Even though every child is a new and unique human being with their own needs and requirements, these elements are very significant, and promoting such interests should enhance the child’s overall well-being.

As already mentioned, another issue to address concerns a possible conflict with parents in cases where they request nondisclosure. Such conflicts are notoriously difficult to manage in clinical practice. Efforts should be made to prevent these conflicts from the outset, particularly in shared decision-making scenarios, where it is important to explore the parents’ desire to withhold the truth from their child (Taub et al., 2023). Indeed, some cultural contexts may make the practice of concealing the truth ethically more acceptable (Childress and Beauchamp, 2013; Taub et al., 2023). Ideally, a compromise should be sought that still promotes truthfulness, by explaining to parents the possibility that the child may already be aware of their condition, and that disclosing the truth could be more beneficial to them than withholding it. A possible approach to addressing this type of issue could be to involve an ethics committee (Taub et al., 2023), which may provide guidance through formal recommendations on how to proceed. Such a committee might also serve as a mediator, bringing together all interested parties to share their perspectives, identify areas of agreement, and explore potential ways forward.

It is not inconceivable to promote, in these cases, a recommendation model of shared decision-making rather than a free market model. Although it is typically used for choosing medical treatments, the child’s best interests also constitute a medical standard, and therefore the decision to tell or withhold the truth may occur in circumstances where it should be explicitly recommended.

At the same time, research shows that parents often feel left alone in these situations, and that when conflicts between parents and clinicians arise, a collaborative alliance is often lacking, which could otherwise help them adopt effective and mutually acceptable solutions. As noted, clinicians can frequently experience emotional difficulties when sharing prognoses with both parents and children. A crucial aspect in these cases is a gradual disclosure of the truth (Childress and Beauchamp, 2013), to avoid a situation in which death becomes something unexpected. In children with medical complexity, death is often a foreseeable event and, precisely because of the difficulty in determining the exact timing of imminent death, it would be advisable to inform the parents and the child progressively as the child’s condition evolves.

## Conclusions

I analyzed whether the use of the therapeutic privilege in the case of imminent death is in the best interests of the child. To understand this, I examined whether the practice is beneficial and whether it respects the interests of seriously ill children. Even though there is probably no definite answer regarding the use of therapeutic privilege in the case of imminent death, establishing its potential advantages remains problematic. It is still unclear whether the best interests of a child align solely with his or her medical interests, and little is known about children with a situation of medical complexity and their special interests. This suggests that the Best Interests Standard, if applied in a generalized way, risks being inadequate. To avoid this difficulty, the paper has argued for a multimodal and flexible approach, capable of capturing the broader range of children’s interests. While nobody would want that a child lives in a condition of fear and anxiety, considering the avoidance of psychological pain and fear as the standard of the best interests of children represents a very limited vision of their interests. As the analysis has shown, disclosure, especially when adapted to the child’s developmental level and circumstances, can sustain relationships, foster trust, and even provide a sense of meaning and control at the end of life.

This paper does not resolve all the difficulties concerning the best interests standard. The reason for this is that when we make decisions for others, there is always a degree of risk in interpreting their well-being and what they would have wanted for themselves. The case of children is particularly complex because their preferences usually are not well known, and they are not considered stable over time. Our responsibility is to seek the best possible choice, even while accepting that a final, definitive answer may always elude us.

## Data Availability

No datasets were generated or analysed during the current study.

## Footnotes

1 In this sense, “imminent” does not imply suddenness or unexpectedness, as the underlying disease trajectory and fatal outcome are often already known; rather, what changes is the perceived temporal proximity of death.

2 While the term is well established in bioethical literature, it is not universally familiar across all clinical contexts (Shalak et al., 2022). The term “privilege” carries no inherent axiological value; rather, its moral significance depends on the justifiability of the act in specific situations. The practice is also referred to as a “therapeutic exception”. My choice to use the term “privilege” in this context highlights the discretionary authority granted to physicians to act in exceptional circumstances, potentially overriding standard informed consent procedures.

3 The case has been summarized for the purpose of this paper.

4 Destination therapy denotes the use of a device as a permanent solution, rather than a temporary bridge to heart transplantation.

5 Although the authors cannot be entirely certain that these discussions did not actually take place and were simply not documented, several communication barriers reported by the healthcare professionals can still be identified in their paper.

6 It is important to note that, although this appears to be the predominant theoretical orientation of the principle, alternative interpretations do exist. The reason why this principle is frequently associated with a consequentialist perspective is that, in medical ethics, decisions often require careful consideration of outcomes. For an overview see Birchely 2021.

8 Article 13 states: “The child shall have the right to freedom of expression; this right shall include freedom to seek, receive and impart information and ideas of all kinds (…)”.
